# Hybrid Surgery Versus Anterior Cervical Discectomy and Fusion in Multilevel Cervical Disc Diseases: A Meta-Analysis: Erratum

**DOI:** 10.1097/01.md.0000490130.88156.9e

**Published:** 2016-07-29

In the article “Hybrid Surgery Versus Anterior Cervical Discectomy and Fusion in Multilevel Cervical Disc Diseases: A Meta-Analysis”,^1^ which appeared in Volume 95, Issue 21 of *Medicine*, the corresponding author's address was listed incompletely in the original article. The article has since been corrected online.
